# Safety of a Bioactive Polyphenol Dietary Supplement in Pediatric Subjects with Acute Diarrhoea

**DOI:** 10.1155/2015/387159

**Published:** 2015-09-07

**Authors:** Shafiqul A. Sarker, Shamima Sultana, Mark Pietroni, Arthur Dover

**Affiliations:** ^1^Dhaka Hospital, icddr,b, 68 Shaheed Tajuddin Ahmed Sarani, Mohakhali, Dhaka 1212, Bangladesh; ^2^Department of Public Health, South Gloucestershire Council, Gloucestershire, UK; ^3^Aptos Travel Clinic, 9099 Soquel Drive, Aptos, CA 95003, USA

## Abstract

The hematological and clinical chemistry profile for children aged 6 months to 5 years with acute diarrhoea was measured in a double blind clinical trial. Subjects were randomized to the study group (*N* = 44) given a bioactive polyphenol dietary supplement in oral rehydration solution (ORS) or to the control group (*N* = 41) given distilled water as a placebo in ORS twice daily for up to 4 days. All subjects received 10 mg zinc daily for the 4 days in the study. Venous blood was collected for complete blood count, electrolytes, liver function, and creatinine upon enrollment (baseline) and at the end of 4 days (end of study); mean values were compared by 95% confidence intervals. Overall, blood factors measured either remained the same over the 4 days or increased or decreased at the same levels between the two groups during the study period. All values were within accepted ranges for paediatric subjects except serum AST (SGOT), where the mean value of the study group approached the upper bound of the range on day 4 but was comparable to the value of the control group. Consumption of this supplement twice daily for 4 days is safe for children and infants.

## 1. Introduction

Diarrhoeal diseases remain a major threat to human health globally. In the early 1980s, diarrhoeal diseases accounted for about 4.6 million deaths from around 1 billion episodes of illness in children younger than 5 years each year [1]. A decade later, even without significant change in incidence, the number of deaths attributable to diarrhoeal diseases dropped to 3.3 million per year [2]. This reduction in death was attributed primarily to implementation of Oral Rehydration Therapy (ORT) protocols coordinated by the World Health Organization (WHO). The most recent estimates indicate that the number of deaths had been further reduced to 2.5 million [3].

While this downward trend is encouraging, the major burden of diarrhoeal illness is experienced still in the developing world, where children suffer from 6 to 7 episodes per year compared to only one episode for children in developed countries [4]. Factors such as poor water supply and sanitation, lack of education about habits, malnutrition, and HIV-associated immunodeficiency underlie the high incidence of diarrhoeal diseases in the developing nations.

Standard management protocols for diarrhoeal illness involve prevention and management of dehydration with the administration of oral or intravenous rehydration fluids, as appropriate, and continued feeding, including breastfeeding for young infants. Administration of rehydration fluids, while effective at reducing some symptoms associated with acute diarrhoea, does not universally shorten the time to its resolution [5–10]. Supplementing with zinc, a micronutrient missing from many indigenous diets, has been reported to reduce time to diarrhoea resolution by 20% [11–15], which has prompted the WHO and UNICEF to recommend routine administration of zinc supplements for 10 to 14 days in the management of diarrhoea in young children, irrespective of aetiology [16]. However, the successful implementation of this practice in many developing countries has been slow due to economics and time to resolution in longer than desired time periods [17–19].

Reduced activity of the immune system is a possible result of malnutrition [20, 21]. Research has noted that malnourished children are stunted in growth and cognitive development, and are at high risk of morbidities from infection and deaths [22–24]. Supplementing these diets with micronutrients can improve wasting, reduce infections and diarrhoeal episodes, and strengthen intestinal tissue [25]. Plant polyphenols constitute one of the largest and most ubiquitous groups of secondary metabolites that are an integral part of the human diet [26]. Several human trials have reported that polyphenol intake is associated with a reduced risk of cardiovascular diseases, cancers, and neurodegenerative diseases [27–34]. This reduction is attributed to the capability of polyphenols to reduce apoptosis [35], downregulate cytokines, such as tumor necrosis factor-alpha [36], and inhibit activation of mitogen-activated protein kinases and nuclear factor kappa B (NF-*κ*B) [37–41].

Building on this research, LiveLeaf Bioscience (San Carlos, CA, USA) has developed a novel extraction process for polyphenols, LiveXtract, that when administered in a porcine model demonstrated a reparative action of mucosa of the small intestine following inoculation with* E*.* coli* [42]. The objective of this study was to evaluate the safety and tolerability of oral administration of a LiveXtract product in young Bangladeshi subjects with infectious diarrhoea.

## 2. Methods

### 2.1. Bioactive Polyphenol Dietary Supplement

The LiveXtract product used in this study, LifeDrops solution, is an aqueous formulation with a base of epigallocatechin gallate (EGCG) and epicatechin gallate (ECG) extracted of the leaves of green tea (*Camellia sinensis*) and punicalagin-*α* and punicalagin-*β* from the pericarp of the pomegranate (*Punica granatum*) fruit. This mixture mimics the action of immunologically active compounds, which are constituents of a plant's innate immune system, being released as fresh plant tissue is digested. Earlier chemical analysis on fresh plant materials performed by LiveLeaf, Inc. researchers determined that manufacturing processes used to obtain these extracts involve extensive cellular disruption and desiccation, resulting in the natural content of the reactive oxygen species (ROS) in the cytoplasm of these plant materials being reduced significantly. The company's proprietary LiveXtract process involves multiple extractions of plant material, aliquot standardization, thermal cycling, pH adjustments, and adduction to achieve a phenolic-ROS ratio similar to that found in many plant tissues. The result is a highly stable product that mimics reduction-oxidation conditions found within living plant cells to achieve bioactive potential not possible with polyphenols alone. Since animal intestinal tissues and many pathogenic bacteria express enzymes that can efficiently convert this stable phenolic-ROS complex into oxidized phenolic groups at the site of infected or damaged tissue, the LifeDrops solution has the potential for localized high affinity peptide binding for effective modulation of inflammatory signaling and microbial virulence factors. The product contains 0.88 mg of the LiveXtract component per 1 mL of solution.

One LiveXtract product that has shown efficacy in pediatric patients is LifeDrops solution [43]. Evaluation of the safety profile of this solution is an appropriate step in developing treatment strategies for pediatric patients.

### 2.2. Clinical Safety Assessment

This was a single center, randomized, double blind clinical trial (RCT) to evaluate the safety of LifeDrops solution in subjects aged 6 months to 5 years when used as an adjunct to institutional standards of care in subjects with infectious diarrhoea irrespective of aetiologies. The study protocol was approved by both the Research Review Committee (RRC) and Ethical Review Committee (ERC) of the International Centre for Diarrhoeal Disease Research, Bangladesh (icddr,b) and registered (ISRCTN11621877). The study was conducted with the understanding and consent of the subjects' parents or guardians.

The study was performed in the Short Stay Unit (SSU) of the icddr,b Dhaka Hospital in accordance with the guidelines on Good Clinical Practice (GCP) for Trials on Therapeutic products (GCMP Working Party on Efficacy on Medicinal Products, Commission on European Communities; Brussels 1991). Parents/guardians of the children were informed on the purpose and nature of the study, methods to be followed and their alternatives, potential risks from the study interventions and procedures, the benefits, the voluntary nature of participation, confidentiality of information, and rights to withdraw from the study. An easily understandable consent form, written in Bengali, was provided to them to read or read out and explained in the event they were unable to read. Informed consent was obtained before enrolment in the study.

#### 2.2.1. Enrolment

Male subjects, between 6 months and 5 years of age, who presented to the Outpatient Department of the Dhaka Hospital of icddr,b with acute diarrhoea (an episode of 48 hours or less) were screened for enrolment into the study. Only male subjects were included so that urine and stool specimens could be collected with minimal potential for cross-contamination. Subjects were excluded if they exhibited fever (>38°C.) and had bloody stool with or without abdominal pain, clinical signs of coexisting severe acute systemic illness (e.g., meningitis, sepsis, and pneumonia), underlying severe chronic disease (e.g., heart disease, cystic fibrosis, diabetes, and liver/kidney malfunction), severe malnutrition, any signs of internal bleeding or black stool, any signs of drug abuse, food allergy or other chronic gastrointestinal diseases, use of antibiotics or any antidiarrhoeal medication during the previous two weeks, any other condition that the admitting physician believed would place the subject at risk if enrolled, those whose stool cultures yielded* Shigella* spp. and cases in which the parent/guardian was unable or unwilling to provide consent.

Upon enrolment, demographic and current illness data including onset and duration of diarrhoea and characteristic of stools were recorded, before presenting to the unit.

#### 2.2.2. Sample Size

This was a pilot safety portion of a therapeutic study, akin to a Phase I drug study [44], to be conducted in subjects in four different age groups: 6 to 23 months, 2 to 5 years, 5 to 8 years, and 18–60 years. The sample size was calculated for 80% power and a significance level of 0.05 to detect a 30% lower duration of diarrhoea in those given the bioactive supplement. Stool output and frequency have been measured at icddr,b for these age groups in the past, along with time to resolution of diarrhoea with standard of care. Administration of LifeDrops solution to address acute diarrhoea has been reported to result in a more than 30% reduction of duration of diarrhoea and stool number in pediatric patients [43] and therefore the intent of a follow-on comparative study was to demonstrate a similar or greater magnitude of reduction. A total of 160 subjects, 40 in each age category, were determined to be an appropriate sample size to be enrolled for statistical significance. Results for teen and adult subjects will be reported in another publication.

#### 2.2.3. Randomization

A random permuted block design was used for allocation of subjects into either the study group (LifeDrops solution added to the ORS) or the control group (distilled water as a placebo added to the ORS). All solutions were prepared in the clinic's pharmacy, the pharmacist being the only person on-site with the randomization key; however, a set of sealed envelopes, each corresponding to the randomization code of the participant, was provided to the investigator to allow immediate unblinding in the event of an emergency that necessitated identification of the product in order to plan clinical management of a participant.

#### 2.2.4. Preparation of the Solutions

The solution provided to a specific subject was prepared in the hospital pharmacy, according to the randomization chart. The volume of LifeDrops solution administered to those in the study arm was based upon the subject's weight (Table 1). Those in the control arm were given the same volume of distilled water with a standard food-coloring agent to match the color of the LifeDrops solution. The solutions were sent to the unit where clinic staff poured it into a sipper cup and then added ORS to total 25 mL of combined liquids.

#### 2.2.5. Administration of the Solutions

The solution was fed under close supervision of a nurse over a period 5 to 10 minutes. Afterward, the sipper cup was rinsed with an additional 10 mL of hypoosmolar ORS and fed to the subject. In the event of vomiting within 15 minutes of feeding either of the solutions, another cup was prepared with either the test or placebo solution and fed again to the subject. Both active and placebo solutions were given 2 times a day, at 08:30 and again at 14:30, for 4 days.

Supplementation with zinc is standard of care for children with diarrhoea at icddr,b. All subjects received a 20 mg tablet of zinc once each day for the 4 days during the study; upon release from the hospital, subjects were given additional 6 tablets in order to complete the institution's standard regime of 10 tablets over 10 days.

#### 2.2.6. Laboratory Studies

A fresh stool specimen was obtained from each subject after enrolment and examined to determine presence of* V. cholera*, rotavirus,* E. coli* (enterotoxigenic, enteropathogenic, and enterohaemorragic),* Shigella* spp., and intestinal protozoa (e.g.,* Giardia lamblia*,* Entamoeba histolytica*, and* Cryptosporidium*). Presence of these pathogens was not an exclusion factor but did assist with further management decisions.

Upon enrolment, 2.5 mL of venous blood was collected under aseptic precautions in EDTA-containing vacutainer tubes for complete blood count, electrolytes, liver (ALT and AST), and creatinine to document baseline values. A second collection of blood was taken at the end of day 4 and the same parameters were tested (end of study).

#### 2.2.7. Monitoring

Each of the participating children was monitored closely for occurrence of adverse events, including gastrointestinal symptoms such as worsening of diarrhoea (increased frequency and volume) or increased frequency of vomiting. Events were noted as to severity and duration, as well as any treatment provided to resolve the event. Vital signs (pulse, respiration, and temperature) and blood pressure were measured thrice daily.

Upon admission, those with severe dehydration received correction with intravenous infusion and those with some dehydration were given oral administration of ORS. During the study, additional ORS volumes were administered as needed for replacement of ongoing loss of stool and the volume provided daily was noted. Breastfeeding* ad libitum* was continued for breastfed infants and children; formula milk for nonbreastfed infants and semisolid or solid local food for children appropriate for age were provided during the 4 days of monitoring.

#### 2.2.8. Statistical Analyses

Mean values and standard deviation were calculated for subject demographic variables, disease history, and enteropathogen presence, with mean differences tested with Student's *t*-test and Chi-square and *Z*-test analyses performed on proportions. Comparison between baseline and end of study laboratory values was determined by 95% confidence intervals. Analyses were performed using MedCalc 13 (MedCalc Software bvba, Ostend, Belgium).

## 3. Results

### 3.1. Admission Characteristics of Subjects

A total of 85 paediatric subjects were enrolled in this safety study, with 44 subjects receiving the LifeDrops solution mixed with ORS and 41 subjects receiving distilled water with ORS. There were no differences in the demographic features of subjects in the 2 groups (Tables 2 and 3). At admission, the subjects in both groups were comparable in terms of age, weight, and length of time they had experienced diarrhoea before coming to the clinic. Overall, more enteropathogens were noted from stool cultures in subjects younger than 2 years versus those 2 to 5 years of age, but between the 2 groups, no pathogen was more prominent. Rotavirus was the most frequent pathogen isolated in both age groups.

### 3.2. Safety of Consumption of LifeDrops Solution

A total of 44 infants and children were administered a volume of LifeDrops solution twice daily for up to 4 days with no adverse events or detrimental physiological outcomes.

During the 4 days of data collection, subjects were given additional ORS as needed to maintain hydration; this was in addition to the 25 mL of ORS administered twice daily to provide either the LifeDrops solution or distilled water (placebo). The mean total volume of ORS administered daily was comparable between the 2 arms.

Vital signs for subjects in these two arms did not differ significantly over the course of the 4 days. The mean blood pressure, temperatures, radial pulse rates, and rates of respiration were comparable among the children in both ages groups (Tables 4 and 5).

### 3.3. Laboratory Testing Analysis

Analysis of blood samples from subjects 5 years of age and younger found no difference in the trends of changes in these parameters among those given LifeDrops and those only consuming ORS (Tables 6 and 7). In the younger subjects (aged 6 to 23 months) the baseline values were not significantly different between the 2 groups at day 0, meaning that subjects in both groups were not different at the start of the monitoring period (Table 6). Subjects in the study group had significantly increased carbon dioxide and significantly decreased levels of sodium, chloride, anion gap, and creatinine by day 4 compared to day 1, likely due to the resolution of dehydration associated with diarrhoeal episodes (Table 6). Those in the control group also had significantly increased carbon dioxide and decreased levels of sodium, chloride, and serum creatinine at day 4 but in addition experienced a significant increase in serum AST (SGOT) (Table 6).

Similarly for subjects 2 to 5 years of age, the values on day 0 for subjects to be given LifeDrops in ORS were not significantly different from the baseline values measured in subjects who received ORS and distilled water (Table 7). Values on day 0 were not significantly different from those found on day 4, except for increases in the percentages of lymphocytes and eosinophils and carbon dioxide. At the same time the total count of neutrophils and serum anion gap and serum creatinine decreased significantly (*p* < 0.05) in both groups. These changes reflect improved renal function and were comparable between both groups.

Overall, the blood factors measured either remained the same or increased or decreased at the same levels in both groups over the 4 days in which either LifeDrops solution in ORS or the placebo in ORS was consumed. Even with increases or decreases from baseline, all values measured were within accepted ranges for pediatric subjects, except for serum AST (SGOT). Reported typical lab values for AST range from 8 to 60 units/L and those in the study group approached the upper bound of that range, with a mean value of 59.2 units/L on day 4. The increase by day 4 was not significant (*p* = 0.06) and that value was not significantly higher than the mean value of 51.4 units/L measured in the control group on day 4 (*p* = 0.07). Since the value at day 4 was not significantly different from the value on day 0, consumption of LifeDrops solution in this group of subjects likely did not contribute to this increase. In contrast, those in the control group realized a highly significant increase in AST (SGOT) from 42.8 units/L on day 0 to 51.4 units/L on day 4 (*p* = 0.007).

## 4. Discussion

This study demonstrates that a bioactive polyphenol solution provided as a supplement to the standard of care for acute diarrhea is safe. No adverse events related to consumption of the supplement or variations in hematology or serum chemistry were observed.

Subjects administered the LifeDrops solution had blood, electrolyte, renal, and liver enzyme values comparable to values of subjects in the control arm, both at the start of monitoring and after consuming the test solution for 4 days. Patients who were dehydrated typically have elevated levels of sodium and chloride and depressed levels of potassium due to loss of fluids with diarrhoea. Chloride values were elevated for subjects in both arms at the start of monitoring, but the values remained within the typical range of values and were lower on day 4 than at the start of monitoring. Subjects in this study were comparatively low in potassium throughout the study period, another sign of fluid losses, but not sufficiently low to require administration of potassium for correction. Renal function was not impaired, according to the value for creatinine, at the end of day 4.

Consumption of polyphenols in excess has been associated with hepatic and renal toxicities, but at levels substantially higher than the amount of polyphenols in LiveXtract solutions [45–48]. Acute doses of a green tea extract administered to Wistar rats through oral gavage at levels of 2000 mg 90% EGCG/kg body weight (bw) were lethal, while doses of 200 mg 90% EGCG/kg body weight did not induce toxicity [49]. In other researches, an LD_50_ of >2,170 mg EGCG/kg bw was calculated for mice [50], and while the specifics of this internal study are not readily available, this is the most widely cited one in product safety documents. A no-observed-adverse-effect level (NOAEL) for another formulation of green tea extract has been estimated to be greater than 2,500 mg/kg bw in mice [51]. The estimate was derived from a subchronic study that administered 2,500 mg/kg bw/day green tea extract by oral gavage for 28 days with no mortality or signs of toxicity. Since the NOAEL was 2,500 mg, the LD_50_ for this particular formulation can be assumed to be much higher. The maximum tolerated dose determined in an 8-week clinical trial of 49 cancer patients was noted to be 4.2 g/m^2^/day or 1.0 g/m^2^/3x day, which is equivalent to consuming seven to eight 120 mL cups of green tea 3 times each day [52], a volume considerably higher than the dietary supplement administered in this study.

Similarly, extractions of pomegranate fruit have a wide safety profile. The oral LD_50_ in an acute toxicity study using Wistar rats and Swiss albino mice was calculated to be >5 g/kg bw [48]. This LD_50_ of >5 g/kg bw (30% punicalagins) translates to 350,000 mg for a 70 kg adult and a subchronic NOAEL of >600 mg/kg bw/day. The concentrations present in LifeDrops dietary supplement (maximum 0.88 mg/day) are of at least 3 orders of magnitude lower than the amounts reported to cause negative health effects in these published studies. Toxicology studies with LiveXtract solutions were negative to differences between recommended serving sizes and 500 times the recommended size (LiveLeaf internal data), which correlates with its use in over 500 humans subjects in the past 3 years with no reports of adverse events in both pediatric and adult patients.

Advances in treatment strategies for diarrhoea have reduced mortality substantially, but it is sobering that, in 2011, there still were 700,000 children under the age of five who died from complications related to diarrhoea [53]. In conditions that promote gastrointestinal stress, providing nutritional support and supporting the immune response of children and infants to microbial assault remain the primary goals of dietary supplements.

## 5. Conclusion

Consumption of LifeDrops solution twice a day for 4 days is safe for children and infant subjects based upon the absence of adverse events and the results of clinical monitoring blood sampling of hematology, electrolyte, hepatic, and renal function factors in subjects 5 years of age and younger.

## Figures and Tables

**Table 1:** Serving sizes of LifeDrops solution or distilled water provided to subjects in either the study or control groups.

Age	Weight (kg)	Serving size (mL)	
2 to 5 years	20 < 30	7	Diluted in
	10 < 20	3.5	25 mL
6 to 23 months	<10	3	ORS

**Table 2:** Admission characteristics of subjects aged 6 to 23 months.

Characteristic	ORS + LifeDrops *N* = 22	ORS + water *N* = 23
Age (months)		
Mean (a)	11	11
Weight (kg)		
Mean (a)	8.6	9
Radial pulse (per min)		
Mean (a)	130	131
Rectal temperature (°C)		
Mean (a)	37.2	37.2
Respiration rate (per min)		
Mean (a)	31	31
Blood pressure (mmHg)		
Mean systolic (a)	90	92
Mean diastolic (a)	61	61
Duration of diarrhoea (hrs)		
(in past 24 hours)		
Mean (a)	26	30
History of vomiting (%) (b)	100%	91%
Frequency of emesis (in past 24 hrs)		
Mean (a)	7	8
Enteropathogens [number of cases (%)]		
Enteroaggregative *E. coli* (c)	10 (45%)	10 (43%)
Enteropathogenic *E. coli* (c)	3 (14%)	2 (9%)
Enterotoxigenic *E. coli* (c)	3 (14%)	2 (9%)
*Shigella* spp. (c)	1 (4%)	0
*Vibrio cholera* (c)	0	1 (4%)
Rotavirus (c)	20 (91%)	17 (74%)
No viral or bacterial		
enteropathogen isolated (c)	1 (4%)	2 (9%)

(a) No significant difference using Student's *t*-test.

(b) No significant difference using Chi-squared test.

(c) No significant difference using two-sample *Z*-test.

**Table 3:** Admission characteristics of subjects aged 2 to 5 years.

Characteristic	ORS + LifeDrops *N* = 22	ORS + water *N* = 18
Age (months)		
Mean (a)	34	37
Height (cm)		
Mean (a)	90.1	90.5
Weight (kg)		
Mean (a)	11.3	12.4
Radial pulse (per min)		
Mean (a)	125	128
Rectal temperature (°C)		
Mean (a)	36.8	36.8
Respiration rate (per min)		
Mean (a)	31	30
Blood pressure (mmHg)		
Mean systolic (a)	96	93
Mean diastolic (a)	65	64
Duration of diarrhoea (hrs)		
(in past 24 hours)		
Mean (a)	30	27
History of vomiting (%) (b)	95.5%	83.3%
Frequency of emesis (in past 24 hrs)		
Mean (a)	9	7
Enteropathogens [number of cases (%)]		
Enteroaggregative *E. coli* (c)	3 (14%)	0
Enteropathogenic *E. coli* (c)	1 (4%)	0
Enterotoxigenic *E. coli *(c)	1 (4%)	2 (11%)
*Shigella* spp. (c)	0	0
*Vibrio cholera* (c)	2 (9%)	1 (5%)
Rotavirus (c)	8 (36%)	8 (44%)
No viral or bacterial		
enteropathogen isolated (c)	8 (36%)	7 (39%)

(a) No significant difference using Student's *t*-test.

(b) No significant difference using Chi-squared test.

(c) No significant difference using two-sample *Z*-test.

**Table 4:** Mean blood pressures, rectal temperatures, pulse rates, and respiration rates over 4 days of subjects 6 to 23 months old consuming either LifeDrops solution in ORS or water (as a placebo) in ORS. Measurements were taken three times each day.

	Day 1			Day 2			Day 3			Day 4		
	6 hr	12 hr	18 hr	6 hr	12 hr	18 hr	6 hr	12 hr	18 hr	6 hr	12 hr	18 hr
Mean blood pressure (mmHg)												
LifeDrops + ORS (systolic/diastolic)		94/63			92/62			93/74			94/64	
Placebo + ORS (systolic/diastolic)		90/63			91/64			91/64			91/64	
Mean rectal temperature (°C.)												
LifeDrops + ORS	37	37	36.9	36.8	36.9	36.7	36.7	36.9	36.7	36.6	36.8	36.9
Placebo + ORS	36.4	37	36.9	36.8	36.9	36.8	36.8	36.8	36.8	36.8	36.6	36.6
Mean pulse rate (beats/min)												
LifeDrops + ORS	130	130	129	127	127	127	128	128	127	127	128	127
Placebo + ORS	130	130	128	128	127	127	128	127	128	127	127	127
Mean respiration rate (breaths/min)												
LifeDrops + ORS	31	30	29	30	29	29	29	29	29	29	29	28
Placebo + ORS	30	30	30	30	29	29	30	30	29	30	29	29

**Table 5:** Mean blood pressures, rectal temperatures, pulse rates, and respiration rates over 4 days of subjects 2 to 5 years old consuming either LifeDrops solution in ORS or water (as a placebo) in ORS. Measurements were taken three times each day.

	Day 1			Day 2			Day 3			Day 4		
	6 hr	12 hr	18 hr	6 hr	12 hr	18 hr	6 hr	12 hr	18 hr	6 hr	12 hr	18 hr
Mean blood pressure (mmHg)												
LifeDrops + ORS (systolic/diastolic)		93/62			93/62			94/61			Not recorded	
Placebo + ORS (systolic/diastolic)		93/65			95/66			96/65			Not recorded	
Mean rectal temperature (°C.)												
LifeDrops + ORS	36.9	36.8	36.8	36.6	36.6	36.6	36.7	36.8	36.8	36.7	36.6	36.4
Placebo + ORS	36.9	37.1	36.6	36.7	36.8	36.8	36.8	36.6	36.7	36.7	36.6	36.7
Mean pulse rate (beats/min)												
LifeDrops + ORS	126	126	127	125	127	127	126	126	126	126	121	121
Placebo + ORS	125	126	124	125	125	124	126	124	125	125	123	122
Mean respiration rate (breaths/min)												
LifeDrops + ORS	29	30	29	29	29	29	30	31	31	30	28	29
Placebo + ORS	31	31	29	31	30	30	31	30	31	30	31	29

**Table 6:** Results from testing of blood samples for various factors in patients aged 6 to 23 months.

	Pediatric normal values^*∗*^	LifeDrops + ORS		Distilled water + ORS	
		*N* = 21		*N* = 23	
		Baseline	End of study	Baseline	End of study
		95% CI	95% CI	95% CI	95% CI
Blood total haematocrit (%)	33 to 42	31.4–33.4	Not reported	31.1–33.6	Not reported
Blood total count leucocyte (K/mcL)	4 to 11	10.1–14.5	Not reported	10.9–14.3	Not reported
Blood total count Neutrophil (%)	30 to 60	33.9–49.4	Not reported	37.2–51.7	Not reported
Blood total count Lymphocyte (%)	25 to 50	39.1–55.3	Not reported	37.6–50.8	Not reported
Total eosinophil (%)	0 to 6	0.46–1.73	Not reported	0.9–3.2	Not reported
Sodium (mmol/L)	135 to 145	136.7–139.5	134.5–136.6	135.4–142.4	133.6–135.9
Potassium (mmol/L)	3.5 to 6.3	4.1–4.6	4.2–4.6	4.1–4.5	3.9–4.5
Chloride (mmol/L)	95 to 112	108.4–112.8	104.0–107.0	107.4–114.2	104.4–107.1
Total carbon dioxide (mEq/L)	18 to 27	14.7–18.2	19.7–23.6	15.3–18.7	18.4–20.9
Serum anion gap (mEq/L)	4 to 20	14.4–16.4	11.8–13.9	13.5–16.3	12.5–14.7
Serum creatinine (mmol/L)	14 to 34	22.9–26.4	18.7–21.9	21.7–26.7	18.8–22.2
Serum ALT (SGPT) (U/L)	12 to 78	23.2–34.1	25.4–38.2	17.9–29.8	22.1–31.7
Serum AST (SGOT) (U/L)	8 to 60	42.8–52.8	50.8–67.6	38.1–47.4	47.1–55.8

^*∗*^Mayo Clinic pediatric test reference values.

**Table 7:** Results from testing of blood samples for various factors in patients aged 2 to 5 years.

	Pediatric normal values^*∗*^	LifeDrops + ORS		Distilled water + ORS	
		*N* = 22		*N* = 18	
		Baseline	End of study	Baseline	End of study
		95% CI	95% CI	95% CI	95% CI
Blood total haematocrit (%)	33 to 42	31.9–37.0	33.2–36.2	32.9–35.7	26.8–32.6
Blood total count Leucocyte (K/mcL)	4 to 11	7.8–11.7	7.8–11.4	9.3–12.5	7.8–19.3
Blood total count Neutrophil (%)	30 to 60	58.8–70.4	27.3–53.5	48.1–61.4	21.8–50.6
Blood total count Lymphocyte (%)	25 to 50	12.6–29.6	32.8–56.1	27.9–39.5	26.6–62.6
Total eosinophil (%)	0 to 6	0.1–0.5	3.2–12.5	0.3–3.3	3.4–16.3
Sodium (mmol/L)	135 to 145	133.4–136.7	135.1–137.2	133.4–136.5	136.0–137.8
Potassium (mmol/L)	3.5 to 6.3	3.4–4.0	3.6–4.2	3.6–4.2	3.2–3.9
Chloride (mmol/L)	95 to 112	104.3–106.8	102.6–105.3	102.7–107.3	104.1–107.0
Total carbon dioxide (mEq/L)	18 to 27	15.6–19.3	21.3–24.5	15.1–19.3	20.4–23.2
Serum anion gap (mEq/L)	4 to 20	14.2–17.4	12.4–14.3	14.9–19.7	12.1–14.6
Serum creatinine (mmol/L)	14 to 34	24.8–29.6	19.8–22.9	23.7–31.9	20.1–24.8
Serum ALT (SGPT) (U/L)	12 to 78	17.1–23.8	19.6–26.5	18.9–30.8	19.6–33.2
Serum AST (SGOT) (U/L)	8 to 60	33.7–41.5	36.2–48.1	36.1–50.5	33.8–45.4

^*∗*^Mayo Clinic Pediatric test reference values.

## References

[1] Snyder J. D., Merson M. H. (1982). The magnitude of the global problem of acute diarrhoeal disease: a review of active surveillance data. *Bulletin of the World Health Organization*.

[2] Bern C., Martines J., De Zoysa I., Glass R. I. (1992). The magnitude of the global problem of diarrhoeal disease: a ten-year update. *Bulletin of the World Health Organization*.

[3] Victora C. G., Bryce J., Fontaine O., Monasch R. (2000). Reducing deaths from diarrhoea through oral rehydration therapy. *Bulletin of the World Health Organization*.

[4] Santosham M., Keenan E. M., Tulloch J., Broun D., Glass R. (1997). Oral rehydration therapy for diarrhea: an example of reverse transfer of technology. *Pediatrics*.

[5] Patel A., Dibley M. J., Mamtani M., Badhoniya N., Kulkarni H. (2009). Zinc and copper supplementation in acute diarrhea in children: a double-blind randomized controlled trial. *BMC Medicine*.

[6] CHOICE Study Group (2001). Multicenter, randomized, double-blind clinical trial to evaluate the efficacy and safety of a reduced osmolarity oral rehydration salts solution in children with acute watery diarrhea. *Pediatrics*.

[7] Khan A. M., Sarker S. A., Alam N. H., Hossain M. S., Fuchs G. J., Salam M. A. (2005). Low osmolar oral rehydration salts solution in the treatment of acute watery diarrhoea in neonates and young infants: a randomized, controlled clinical trial. *Journal of Health, Population and Nutrition*.

[8] Atia A. N., Buchman A. L. (2009). Oral rehydration solutions in non-cholera diarrhea: a review. *American Journal of Gastroenterology*.

[9] Munos M. K., Walker C. L. F., Black R. E. (2010). The effect of oral rehydration solution and recommended home fluids on diarrhoea mortality. *International Journal of Epidemiology*.

[10] Sentongo T. A. (2004). The use of oral rehydration solutions in children and adults. *Current Gastroenterology Reports*.

[11] Bhatnagar S., Bahl R., Sharma P. K., Kumar G. T., Saxena S. K., Bhan M. K. (2004). Zinc with oral rehydration therapy reduces stool output and duration of diarrhea in hospitalized children: a randomized controlled trial. *Journal of Pediatric Gastroenterology and Nutrition*.

[12] Roy S. K., Tomkins A. M., Akramuzzaman S. M. (1997). Randomised controlled trial of zinc supplementation in malnourished Bangladeshi children with acute diarrhoea. *Archives of Disease in Childhood*.

[13] Bahl R., Bhandari N., Saksena M. (2002). Efficacy of zinc-fortified oral rehydration solution in 6- to 35-month-old children with acute diarrhea. *Journal of Pediatrics*.

[14] Boran P., Tokuc G., Vagas E., Oktem S., Gokduman M. K. (2006). Impact of zinc supplementation in children with acute diarrhoea in Turkey. *Archives of Disease in Childhood*.

[15] Sachdev H. P., Mittal N. K., Mittal S. K., Yadav H. S. (1988). A controlled trial on utility of oral zinc supplementation in acute dehydrating diarrhea in infants. *Journal of Pediatric Gastroenterology and Nutrition*.

[16] Fontaine O. (2006). Zinc and treatment of diarrhoea. *Medecine Tropicale*.

[17] Patel A. B., Badhoniya N., Dibley M. J. (2013). Zinc and copper supplementation are not cost-effective interventions in the treatment of acute diarrhea. *Journal of Clinical Epidemiology*.

[18] Polat T. B., Uysalol M., Çetinkaya F. (2003). Efficacy of zinc supplementation on the severity and duration of diarrhea in malnourished Turkish children. *Pediatrics International*.

[19] Wadhwa N., Natchu U. C. M., Sommerfelt H. (2011). ORS containing zinc does not reduce duration or stool volume of acute diarrhea in hospitalized children. *Journal of Pediatric Gastroenterology and Nutrition*.

[20] Shankar A. H., Prasad A. S. (1998). Zinc and immune function: the biological basis of altered resistance to infection. *The American Journal of Clinical Nutrition*.

[21] Kaminogawa S., Nanno M. (2004). Modulation of immune functions by foods. *Evidence-Based Complementary and Alternative Medicine*.

[22] Wierzba T. F., El-Yazeed R. A., Savarino S. J. (2001). The interrelationship of malnutrition and diarrhea in a periurban area outside Alexandria, Egypt. *Journal of Pediatric Gastroenterology and Nutrition*.

[23] Mahgoub H. M., Adam I. (2012). Morbidity and mortality of severe malnutrition among Sudanese children in New Halfa Hospital, Eastern Sudan. *Transactions of the Royal Society of Tropical Medicine and Hygiene*.

[24] Caulfield L. E., de Onis M., Blössner M., Black R. E. (2004). Undernutrition as an underlying cause of child deaths associated with diarrhea, pneumonia, malaria, and measles. *The American Journal of Clinical Nutrition*.

[25] Mda S., van Raaij J. M. A., de Villiers F. P. R., Kok F. J. (2013). Impact of multi-micronutrient supplementation on growth and morbidity of HIV-infected South African children. *Nutrients*.

[26] Manach C., Scalbert A., Morand C., Rémésy C., Jiménez L. (2004). Polyphenols: food sources and bioavailability. *The American Journal of Clinical Nutrition*.

[27] Zaveri N. T. (2006). Green tea and its polyphenolic catechins: medicinal uses in cancer and noncancer applications. *Life Sciences*.

[28] De Lira Mota K. S., Dias G. E. N., Pinto M. E. F. (2009). Flavonoids with gastroprotective activity. *Molecules*.

[29] Varilek G. W., Yang F., Lee E. Y. (2001). Green tea polyphenol extract attenuates inflammation in interleukin-2-deficient mice, a model of autoimmunity. *Journal of Nutrition*.

[30] Dell'Agli M., Galli G. V., Bulgari M. (2010). Ellagitannins of the fruit rind of pomegranate (*Punica granatum*) antagonize *in vitro* the host inflammatory response mechanisms involved in the onset of malaria. *Malaria Journal*.

[31] Fawole O. A., Makunga N. P., Opara U. L. (2012). Antibacterial, antioxidant and tyrosinase-inhibition activities of pomegranate fruit peel methanolic extract. *BMC Complementary and Alternative Medicine*.

[32] Ismail T., Sestili P., Akhtar S. (2012). Pomegranate peel and fruit extracts: a review of potential anti-inflammatory and anti-infective effects. *Journal of Ethnopharmacology*.

[33] Hernaez A., Fernandez-Castillejo S., Farras M. (2014). Olive oil polyphenols enhance high-density lipoprotein function in humans: a randomize controlled trial. *Arteriosclerosis, Thrombosis, and Vascular Biology*.

[34] Malar D. S., Devi K. P. (2014). Dietary polyphenols for treatment of Alzheimer's disease—future research and development. *Current Pharmaceutical Biotechnology*.

[35] Du G.-J., Zhang Z., Wen X.-D. (2012). Epigallocatechin gallate (EGCG) is the most effective cancer chemopreventive polyphenol in green tea. *Nutrients*.

[36] Romier-Crouzet B., Van de Walle J., During A. (2009). Inhibition of inflammatory mediators by polyphenolic plant extracts in human intestinal Caco-2 cells. *Food and Chemical Toxicology*.

[37] Rasheed Z., Akhtar N., Anbazhagan A. N., Ramamurthy S., Shukla M., Haqqi T. M. (2009). Polyphenol-rich pomegranate fruit extract (POMx) suppresses PMACI-induced expression of pro-inflammatory cytokines by inhibiting the activation of MAP kinases and NF-*κ*B in human KU812 cells. *Journal of Inflammation*.

[38] Xu X., Yin P., Wan C. (2014). Punicalagin inhibits inflammation in LPS-induced RAW264.7 macrophages via the suppression of TLR4-mediated MAPKs and NF-*κ*B activation. *Inflammation*.

[39] Kim H.-S., Quon M. J., Kim J.-A. (2014). New insights into the mechanisms of polyphenols beyond antioxidant properties: lessons from the green tea polyphenol, epigallocatechin 3-gallate. *Redox Biology*.

[40] Khan N., Syed D. N., Pal H. C., Mukhtar H., Afaq F. (2012). Pomegranate fruit extract inhibits UVB-induced inflammation and proliferation by modulating NF-*κ*B and MAPK signaling pathways in mouse skin. *Photochemistry and Photobiology*.

[41] Ahmed S., Wang N., Hafeez B. B., Cheruvu V. K., Haqqi T. M. (2005). Punica granatum L. extract inhibits IL-1*β*-induced expression of matrix metalloproteinases by inhibiting the activation of MAP kinases and NF-*κ*B in human chondrocytes in vitro. *Journal of Nutrition*.

[42] Bontempo V., Jiang X. R., Cheli F. (2014). Administration of a novel plant extract product via drinking water to post-weaning piglets: effects on performance and gut health. *Animal*.

[43] Dover A., Patel N., Park K. T. (2015). Rapid cessation of acute diarrhea using a novel solution of bioactive polyphenols: a randomized trial in Nicaraguan children. *PeerJ*.

[44] Storer B. E. (1989). Design and analysis of Phase I clinical trials. *Biometrics*.

[45] Inoue H., Akiyama S., Maeda-Yamamoto M., Nesumi A., Tanaka T., Murakami A. (2011). High-dose green tea polyphenols induce nephrotoxicity in dextran sulfate sodium-induced colitis mice by down-regulation of antioxidant enzymes and heat-shock protein expressions. *Cell Stress and Chaperones*.

[46] Mennen L. I., Walker R., Bennetau-Pelissero C., Scalbert A. (2005). Risks and safety of polyphenol consumption. *The American Journal of Clinical Nutrition*.

[47] Vlachojannis C., Zimmermann B. F., Chrubasik-Hausmann S. (2015). Efficacy and safety of pomegranate medicinal products for cancer. *Evidence-Based Complementary and Alternative Medicine*.

[48] Patel C., Dadhaniya P., Hingorani L., Soni M. G. (2008). Safety assessment of pomegranate fruit extract: acute and subchronic toxicity studies. *Food and Chemical Toxicology*.

[49] Isbrucker R. A., Edwards J. A., Wolz E., Davidovich A., Bausch J. (2006). Safety studies on epigallocatechin gallate (EGCG) preparations. Part 2: dermal, acute and short-term toxicity studies. *Food and Chemical Toxicology*.

[50] Rinsho K. T. (1960). *Clinical Report*.

[51] Hsu Y.-W., Tsai C.-F., Chen W.-K., Huang C.-F., Yen C.-C. (2011). A subacute toxicity evaluation of green tea (*Camellia sinensis*) extract in mice. *Food and Chemical Toxicology*.

[52] Pisters K. M. W., Newman R. A., Coldman B. (2001). Phase I trial of oral green tea extract in adult patients with solid tumors. *Journal of Clinical Oncology*.

[53] Das J. K., Salam R. A., Bhutta Z. A. (2014). Global burden of childhood diarrhea and interventions. *Current Opinion in Infectious Diseases*.

